# Significance of fatty liver index to detect prevalent ischemic heart disease: evidence from national health and nutrition examination survey 1999–2016

**DOI:** 10.3389/fcvm.2023.1171754

**Published:** 2023-10-12

**Authors:** Yuyu Niu, Guifang Wang, Xianjun Feng, Hongyi Niu, Wenrui Shi

**Affiliations:** ^1^ Department of Cardiovascular Medicine, First People's Hospital of Xinxiang City and The Fifth Affiliated Hospital of Xinxiang Medical College, Xinxiang, China; ^2^ Sanquan College, Xinxiang Medical University, Xinxiang, China; ^3^ Department of Cardiology, Shanghai Chest Hospital, Shanghai Jiao Tong University, Shanghai, China

**Keywords:** epidemiology, NHANES, fatty liver index, ischemic heart disease, general population

## Abstract

**Background:**

Non-alcoholic fatty liver disease (NAFLD) contributes to the development of ischemic heart disease via multiple mechanisms. Fatty liver index (FLI) has been proposed as an accurate, convenient, and economic surrogate of the severity of NAFLD. Our present study aims to assess the association between FLI and the prevalent IHD and to evaluate the potential value of FLI to refine the detection of prevalent IHD in the general population.

**Methods:**

Our work recruited 32,938 subjects from the National Health and Nutrition Examination Survey 1999–2016. IHD was diagnosed according to the subjects’ self-report. FLI was determined based on triglycerides, BMI, *γ*-glutamyltransferase, and waist circumference.

**Results:**

2,370 (7.20%) subjects were diagnosed with IHD. After adjustment of age, sex, race, current smoking, current drinking, PIR, BMI, WC, TC, TG, GGT, Scr, FPG, SBP, anti-hypertensive therapy, anti-diabetic therapy, and lipid-lowering therapy, one standard deviation increase of FLI resulted in a 27.0% increment of the risk of prevalent IHD. In the quartile analysis, we observed a 1.684 times risk of prevalent IHD when comparing the fourth quartile with the first quartile, and there was a trend towards higher risk across the quartiles. The smooth curve fitting displayed a linear relationship between FLI and the presence of IHD without any threshold or saturation effect. Subgroup analysis revealed a robust association in conventional cardiovascular subpopulations, and the association could be more prominent in female subjects and diabetes patients. ROC analysis demonstrated an incremental value of FLI for detecting prevalent IHD after introducing it to conventional cardiovascular risk factors (AUC: 0.823 vs. 0.859, *P* for comparison <0.001). Also, results from reclassification analysis implicated that more IHD patients could be correctly identified by introducing FLI into conventional cardiovascular risk factors (continuous net reclassification index: 0.633, *P* < 0.001; integrated discrimination index: 0.034, *P* < 0.001).

**Conclusion:**

The current analysis revealed a positive and linear relationship between FLI and the prevalent IHD. Furthermore, our findings suggest the incremental value of FLI to refine the detection of prevalent IHD in the general population.

## Introduction

Ischemic heart disease (IHD) has been one of the prominent causes of death globally for decades. The mortality caused by IHD reached 116.9 per 10,000 early in 2017 (1). Under this grim situation, an approach to improve and simplify the detection of subclinical IHD is essential to alleviate the burden of the secondary prevention of IHD.

The presence of non-alcoholic fatty liver disease (NAFLD) is closely associated with an increased risk of IHD (2, 3). From an epidemiological point of view, NAFLD and cardiovascular diseases share several risk factors, including metabolic dysfunction and lifestyle habits (4). Previous studies suggested an association between NAFLD and the risk of several cardiovascular diseases, particularly with IHD (5, 6). Published data have demonstrated that NAFLD is associated with subclinical atherosclerosis and an elevated ten-year IHD risk score independent of diabetes and hypertension (7–10). Furthermore, a recent systemic review, which included 20 studies, has demonstrated that NAFLD patients showed a significantly increased risk of myocardial infarction (11). The pathophysiological mechanism underlying this association is only partially discovered, but it is likely complex and resulting from the interplay of different, bidirectional pathways, including endothelial dysfunction, vascular inflammation, and impaired glucose and lipid metabolism (4). Due to the strong association between NAFLD and IHD, estimating the severity of NAFLD could be a possible approach to benefit the early detection of IHD in the general population. However, the current diagnosis of NAFLD relies on liver ultrasonography, computed tomography, magnetic resonance spectroscopy, and liver biopsy (12); all these methods are costly, inconvenient, and unsuitable for frequent monitoring in primary care conditions. Accordingly, an economical, convenient, and non-invasive method to achieve routine monitoring of NAFLD severity is needed to advance the early identification of IHD in the general population.

Fatty liver index (FLI) was proposed to assess the severity of NAFLD (13). Previous studies have identified its value in predicting several atherosclerotic cardiovascular diseases (14–16). However, evidence regarding the usefulness of FLI in improving the detection of IHD in the general population is still limited. Thus, the present work aims to assess the association between FLI and the prevalent IHD and investigate the potential of FLI to refine the detection of prevalent IHD in a general American population.

## Methods

### Study participants

Our population was derived from the National Health and Nutrition Examination Survey (NHANES) 1999–2016. A detailed description of the NHANES study's protocol and methods is available at its official website (https://wwwn.cdc.gov/nchs/nhanes/ContinuousNhanes/Default.aspx?BeginYear=2013). Briefly, the NHANES survey is conducted by the National Center for Health Statistics (NCHS), a department of the Centers for Disease Control and Prevention (CDC). The NHANES is a continuous cross-sectional survey conducted in America every two years. The survey adopts a multistage, stratified, and clustered probability sampled pattern to maintain its representativity. The primary objective of NHANES is to assess the number and percentage of people with selected diseases and risk factors in the American population. From 1999 to 2016, a total number of 92,062 subjects completed the data collection process. In the current analysis, we included subjects with completed data about the IHD questionnaire, FLI value, and related covariates, and finally included 32,938 subjects. The NCHS institutional Ethics Review Board approved the study protocol. All participants provided written informed consent. All data in the present analysis is accessible to the public at NHANES's official website.

### Measurements

During the data collection process, interviews were performed at the subjects’ homes, while physical and laboratory examinations were conducted in the Mobile Examination Center (MEC). Trained interviewers collected the demographic data with a computer-assisted personal interviewing method. If the subjects could not answer the questions alone, a family member would answer them. Current drinking was determined as having at least 12 drinks in the past year. Current smoking was defined as answering “every day” or “some days” for the question “Do you now smoke cigarettes?”

Anthropometric parameters were measured under the standard protocol. Height and waist circumference (WC) were quantified to the nearest 0.1 cm; weight was quantified to the nearest 0.1 kg. Blood pressure measurement was also performed according to standard operating procedure. After sitting and resting quietly for 5 min, the blood pressure was measured by a calibrated sphygmomanometer. We employed the mean of 3 blood pressure recordings in our analysis. Detailed information about the blood pressure measurement was documented in the “Physician Examination Procedures Manual” on the NHANES official website (https://wwwn.cdc.gov/nchs/nhanes/continuousnhanes/manuals.aspx?BeginYear=2013).

Laboratory tests were conducted at the laboratories certified by NCHS. Detailed information about the laboratory tests was summarized in the official “Laboratory procedures manual” (https://wwwn.cdc.gov/nchs/nhanes/continuousnhanes/manuals.aspx?BeginYear=2013). Briefly, the whole blood count differential used VCS technology, and the Beckman Coulter DXH 800 was used as the hematology analyzer. Blood lipids were quantified by enzymatic assay on the Roche Modular *P* and Roche Cobas 6,000 chemistry analyzers. Fasting plasma glucose was determined by the oxygen rate method on the Modular Chemistry side of the Beckman DxC800. The DxC800 modular chemistry side tested serum creatinine (Scr) through using the Jaffe rate method.

### Definitions

The following standard formula calculated FLI: FLI = [e^0.953^ ^× ln(TG) + 0.139 × BMI + 0.718 × ln(GGT) + 0.053 × WC – 15.745^)]/[1 + e^0.953^ ^× ln(TG) + 0.139 × BMI + 0.718 × ln (GGT) + 0.053 × WC – 15.745^] × 100 (13), TG means triglycerides, BMI stands for body mass index, GGT indicates *γ*-glutamyltransferase, WC refers to waist circumference. Anti-diabetic therapy was defined as using any anti-diabetic medicine in the past two weeks. Diabetes was diagnosed as fasting plasma glucose (FPG) ≥7 mmol/L and / or self-reported anti-diabetic therapy according to the published guideline (17). Anti-hypertensive therapy referred to any blood pressure-lowering medicine intake in the past two weeks. Hypertension was diagnosed as mean systolic blood pressure (SBP) ≥140 mmHg and/or mean diastolic blood pressure ≥90 mmHg; Additionally, subjects with self-reported anti-hypertensive therapy were also recognized as hypertensive patients (18). Lipid-lowering therapy was determined as input of lipid-lowering medicine in the past two weeks. Diagnosis of IHD was identified if the subjects answered “yes” to the question “Ever told you had coronary heart disease? (Questionnaire code: MCQ160c)”, “Ever told you had angina/angina pectoris? (Questionnaire code: MCQ160d)”, or “Ever told you had a heart attack? (Questionnaire code: MCQ160e)”.

### Statistical analysis

Statistical analysis was performed using Stata Statistical Software (version 15.0; StataCorp. LLC. 4905 Lakeway Drive, College Station, Texas 77845 USA) and statistical software packages R (http://www.R-project.org, The R Foundation), EmpowerStats (http://www.empowerstats.com, X&Y Solutions, Inc., Boston, MA). Statistical significance was noted as a two-tailed *P* value < 0.05. Statistical data were weighted according to the survey design of NHANES (https://wwwn.cdc.gov/nchs/nhanes/tutorials/module3.aspx). Continuous variates were expressed as the mean value with 95% confidence intervals (CI). Categorical variates were also summarized as frequency and 95% CI. *T*-test and Chi-square test were performed to compare continuous and categorical variates, respectively. Multivariate logistic regression analysis was conducted to investigate the independent association between FLI and the prevalent IHD. Normalized FLI was generated by a *z*-score change [(FLI-mean of FLI)/SD]. The results of regression analysis were listed as odds ratios (ORs) and 95% confidence intervals (95% CI). To confirm whether the association between FLI and the prevalent IHD was linear in the full range of FLI, we employed a generalized additive model (GAM) with a spline smoothing function, and we also conducted a logarithmic likelihood ratio test to compare one pairwise and two pairwise logistic regression model. Finally, the current study also engaged receiver operating characteristic (ROC) curve and reclassification analysis, including continuous net reclassification index (NRI) and integrated discrimination index (IDI), to assess the potential value of FLI to improve the detection of prevalent IHD.

## Results

Characteristics of the enrolled participants were summarized in Table 1. Among the enrolled 32,938 subjects, 2,370 (7.20%) subjects were detected as IHD patients. Regarding the demographic data, IHD patients were older than non-IHD subjects. Male distribution was significantly higher in the IHD group than in the non-IHD group. IHD patients had a relatively higher percentage of non-Hispanic white than non-IHD subjects. Non-IHD had substantially higher income level (displayed as higher PIR). The non-IHD group had a relatively higher percentage of current drinking status and a relatively lower percentage of current smoking than the IHD group. As for the anthropometric parameters, weight, BMI, WC, and SBP levels were significantly higher in the IHD group. Laboratory indexes like FPG, TG, GGT, and serum creatine (Scr) were substantially higher in the IHD group, and the TC was significantly lower in the IHD group than in the non-IHD group. About medical history characteristics, the IHD group had higher percentages of anti-hypertensive therapy, anti-diabetic therapy, lipid-lowering therapy, diagnosed hypertension, and diagnosed diabetes than the non-IHD group. Finally, the FLI level was significantly higher in the IHD group than in the non-IHD group.

**Table 1:** Data characteristics of enrolled subjects grouped by the presence of IHD.

Variables	Total (*n* = 32,938)	IHD (*n* = 2,370)	non-IHD (*n* = 30,568)	*P* value
Age (years)	46.48 (46.07, 46.89)	64.57 (63.87, 65.27)	45.37 (44.97, 45.76)	<0.001
Male (%)	48.79 (48.31, 49.28)	59.87 (57.02, 62.66)	48.11 (47.58, 48.65)	<0.001
Race (%)				<0.001
Mexican American	8.95 (7.91, 10.12)	5.95 (4.95, 7.14)	9.14 (8.08, 10.32)	
Other Hispanic	6.74 (5.57, 8.12)	3.59 (2.54, 5.04)	6.93 (5.75, 8.33)	
Non-Hispanic White	37.46 (34.92, 40.07)	42.72 (38.55, 46.99)	37.13 (34.63, 39.71)	
Non-Hispanic Black	8.73 (7.85, 9.70)	7.09 (5.87, 8.55)	8.83 (7.93, 9.83)	
Others	38.12 (35.28, 41.05)	40.65 (36.73, 44.70)	37.97 (35.10, 40.92)	
PIR	3.02 (2.95, 3.08)	2.68 (2.57, 2.78)	3.04 (2.97, 3.10)	<0.001
Current smoking (%)	18.07 (17.26, 18.92)	19.83 (17.74, 22.10)	17.97 (17.13, 18.83)	0.094
Current drinking (%)	56.64 (55.00, 58.27)	53.90 (50.70, 57.06)	56.81 (55.15, 58.45)	0.052
Height (cm)	168.99 (168.82, 169.16)	168.35 (167.77, 168.92)	169.03 (168.86, 169.20)	0.982
Weight (kg)	81.83 (81.44, 82.23)	85.15 (84.03, 86.27)	81.63 (81.23, 82.03)	<0.001
BMI (kg/m^2^)	28.57 (28.43, 28.71)	29.91 (29.57, 30.25)	28.49 (28.35, 28.63)	<0.001
WC (cm)	98.08 (97.71, 98.44)	105.12 (104.30, 105.95)	97.64 (97.28, 98.01)	<0.001
SBP (mmHg)	122.19 (121.82, 122.56)	129.82 (128.68, 130.96)	121.72 (121.37, 122.08)	<0.001
DBP (mmHg)	70.85 (70.50, 71.21)	67.23 (66.50, 67.96)	71.08 (70.73, 71.43)	<0.001
FPG (mmol/L)	5.42 (5.39, 5.45)	6.24 (6.11, 6.36)	5.36 (5.34, 5.39)	<0.001
TC (mmol/L)	5.11 (5.09, 5.13)	4.83 (4.76, 4.91)	5.12 (5.10, 5.15)	<0.001
TG (mmol/L)	1.70 (1.67, 1.72)	1.95 (1.87, 2.03)	1.68 (1.65, 1.71)	<0.001
GGT (U/L)	28.08 (27.54, 28.63)	32.25 (30.72, 33.77)	27.83 (27.25, 28.41)	<0.001
Scr (*μ*mol/L)	77.60 (77.14, 78.06)	92.55 (90.24, 94.87)	76.69 (76.23, 77.15)	<0.001
Anti-hypertensive therapy (%)	24.20 (23.35, 25.07)	64.57 (61.96, 67.09)	21.72 (20.95, 22.52)	<0.001
Anti-diabetic therapy (%)	6.46 (6.12, 6.81)	21.38 (19.32, 23.60)	5.54 (5.23, 5.87)	<0.001
Lipid-lowering therapy (%)	14.40 (13.78, 15.04)	54.02 (51.10, 56.92)	11.97 (11.42, 12.54)	<0.001
Hypertension (%)	31.94 (30.98, 32.91)	70.56 (67.97, 73.03)	29.57 (28.65, 30.59)	<0.001
Diabetes (%)	9.96 (9.53, 10.40)	28.15 (25.99, 30.42)	8.84 (8.44, 9.27)	<0.001
FLI	51.35 (50.64, 52.06)	64.36 (62.84, 65.88)	50.55 (49.82, 51.27)	<0.001

Data were summarized as mean (95% confidence intervals) or numbers (95% confidence intervals) according to their data type. IHD, ischemic heart disease; PIR, poverty-income ratio; BMI, body mass index; WC, waist circumference; SBP, systolic blood pressure; DBP, diastolic blood pressure; FPG, fasting plasma glucose; TC, total cholesterol; TG, triglycerides; GGT, *γ*-glutamyltransferase; Scr, serum creatine; FLI, fatty liver index.

Our study employed logistic regression analysis to evaluate the relationship between FLI and the prevalent IHD in our population (Table 2). In the crude model, each SD increase of the normalized FLI was associated with an additional 54.2% risk of the presence of IHD. After adjustment of age, sex, and race, the risk for each SD increase changed to 45.5%. Further adjustment of covariates, including current smoking, current drinking, PIR, BMI, WC, TC, TG, GGT, Scr, FPG, SBP, anti-hypertensive therapy, anti-diabetic therapy, and lipid-lowering therapy, diminished the risk for each SD increase of the normalized FLI to 27.0%. When dividing FLI into quartiles, the top quartile had a 1.684 times risk for the prevalent IHD than the bottom quartile, and the risk for prevalent IHD showed a trend towards a more significant risk across the quartiles (*P* for trend = 0.002).

**Table 2:** Independent association between FLI and the prevalent IHD.

Variables	Odds Ratio (95% CI)					
	Crude	*P* value	Model 1	*P* value	Model 2	*P* value
FLI (Per 1 SD increase)	1.542 (1.463, 1.627)	<0.001	1.455 (1.369, 1.546)	<0.001	1.270 (1.106, 1.458)	0.001
Quartiles of FLI						
Quartile 1	Reference		Reference		Reference	
Quartile 2	2.225 (1.809, 2.735)	<0.001	1.387 (1.124, 1.711)	0.003	1.232 (0.988, 1.538)	0.064
Quartile 3	2.865 (2.399, 3.421)	<0.001	1.727 (1.448, 2.059)	<0.001	1.370 (1.094, 1.716)	0.007
Quartile 4	3.555 (2.955, 4.278)	<0.001	2.611 (2.174, 3.135)	<0.001	1.684 (1.228, 2.310)	0.001
*P* for trend		<0.001		<0.001		0.002

Model 1: age, sex, race. Model 2: Model 1 + current smoking, current drinking, PIR, BMI, WC, TC, TG, GGT, Scr, FPG, SBP, anti-hypertensive therapy, anti-diabetic therapy, and lipid-lowering therapy. FLI, fatty liver index; IHD, ischemic heart disease; CI, confidence interval; SD, standard deviation; PIR, poverty-income ratio; BMI, body mass index; WC, waist circumference; TC, total cholesterol; TG, triglycerides; GGT, *γ*-glutamyltransferase; Scr, serum creatine; FPG, fasting plasma glucose; SBP, systolic blood pressure.

**Figure 1:**
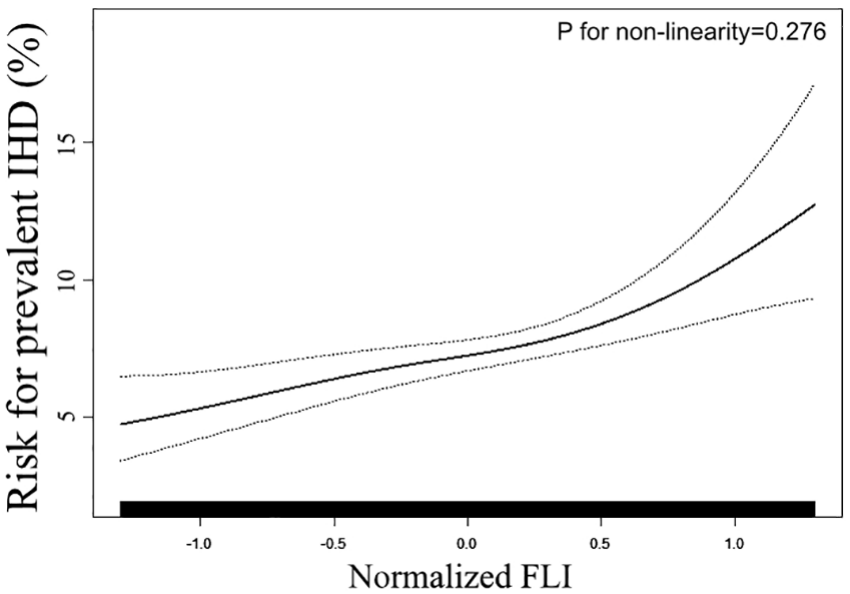
Smooth curve fitting evaluating the association between FLI and the prevent IHD. Smooth curve fitting was conducted through a generalized additive model with the adjustment of all covariates used in Model 2 of Table 2. In the plot, the ratio of prevalent IHD increased linearly with the increment of FLI, suggesting the association between FLI and prevalent LVH was linear in the full range of FLI. FLI: fatty liver index; IHD: ischemic heart disease.

To validate the trend towards a greater risk of prevalent IHD that was observed in the logistic regression analysis, we further conducted a smooth curve fitting. As displayed in Figure 1, the risk of IHD increased proportionally with the increment of Normalized FLI, and we did not observe any threshold or saturation phenomenon in the association between normalized FLI and the prevalent IHD. Consistently, *P* for non-linearity test was insignificant (0.276).

**Figure 2:**
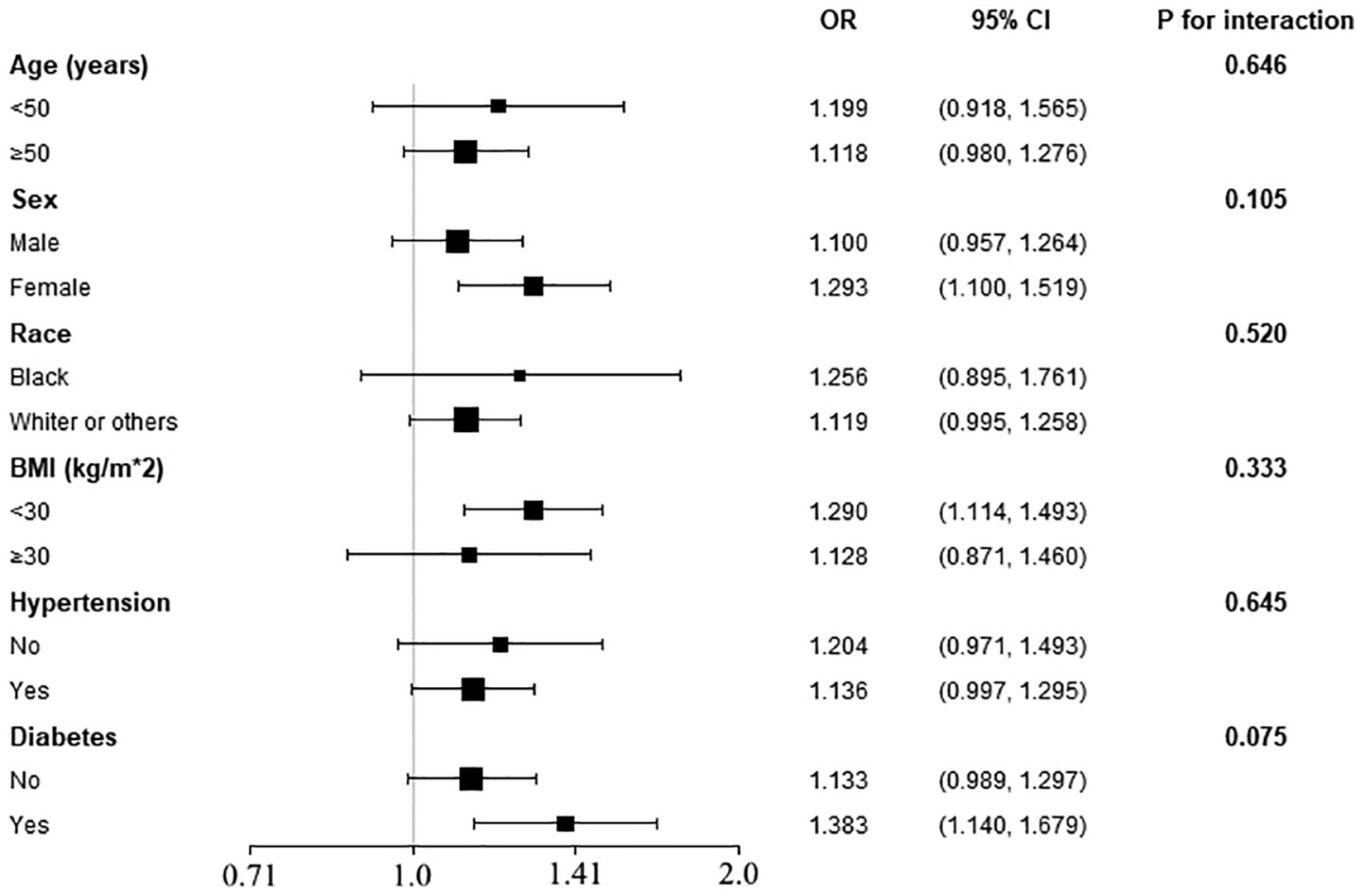
Subgroup analysis of the association between FLI and the prevalent IHD. The models were adjusted for all covariates used in Model 2 of Table 2, except for the variates used to define the stratum. *P* for interaction in all subgroups was insignificant, implicating the association between FLI and IHD was robust in these subpopulations.

To determine the consistency of our main result among common cardiovascular subpopulations, we further employed subgroup analysis (Figure 2). The logistic models were adjusted for all covariates used in Model 2 of Table 2, except those used to define the subgroups. As displayed in Figure 2, the positive association between FLI and the prevalent IHD was also observed in age (<50 or ≥50 years old), sex (male or female), race (black, white, or others), obesity (BMI < 30 kg/m^2^), hypertension (yes or no), and diabetes (yes or no) subgroups, and the interaction effect was insignificant in all these subgroups. However, although the difference was insignificant, the effect size of the association was larger in female subjects and diabetes patients than in male subjects and non-diabetes subjects, respectively.

ROC and reclassification analysis were utilized to investigate the potential usefulness of FLI to improve the detection of prevalent IHD in our population (Table 3). Regarding the results of ROC analysis, although the AUC of FLI itself was limited, we still observed a significant improvement of the AUC (0.823 vs. 0.859, *P* for comparison <0.001) when introducing FLI into clinical risk factors (including age, sex, race, current smoking, current drinking, PIR, BMI, WC, TC, TG, GGT, Scr, FPG, SBP, anti-hypertensive therapy, anti-diabetic therapy, lipid-lowering therapy). Moreover, continuous NRI (0.633, *P* < 0.001) and IDI (0.034, *P* < 0.001) in the reclassification analysis also supported the usefulness of FLI in improving the detection of prevalent IHD.

**Table 3:** Assessment of the value of FLI for detecting prevalent IHD.

Model	AUC (95% CI)	*P* value	*P* for comparison	NRI (continuous)	*P* value	IDI	*P* value
FLI	0.603 (0.598, 0.609)	<0.001		–	–	–	–
Clinical risk factors^a^	0.823 (0.818, 0.827)	<0.001	<0.001	–	–	–	–
Clinical risk factors + FLI	0.859 (0.855, 0.862)	<0.001		0.633 (0.593, 0.673)	<0.001	0.034 (0.031, 0.038)	<0.001

^a^ Clinical risk factors: age, sex, race, current smoking, current drinking, PIR, BMI, WC, TC, TG, GGT, Scr, FPG, SBP, anti-hypertensive therapy, anti-diabetic therapy, and lipid-lowering therapy. FLI, fatty liver index; IHD, ischemic heart disease; AUC, area under the curve; NRI, net reclassification index; IDI, integrated discrimination index; PIR, poverty-income ratio; BMI, body mass index; WC, waist circumference; TC, total cholesterol; TG, triglycerides; GGT, *γ*-glutamyltransferase; Scr, serum creatine; FPG, fasting plasma glucose; SBP, systolic blood pressure.

## Discussion

The current analysis discovered a positive association between FLI and the prevalent IHD in a representative American population. Furthermore, the association was nearly linear in the whole range of the FLI, indicating the ratio of prevalent IHD increases proportionally with the elevation of FLI. Moreover, the association was consistent in several conventional cardiovascular subpopulations, and the effect size was potentially larger in female subjects and diabetes patients. Additionally, both ROC and reclassification analysis supported the potential usefulness of FLI to improve the detection of prevalent IHD in the general population. In general, FLI may serve as a linear indicator with economic, convenient, and non-invasive characteristics to refine the detection of prevalent IHD in the general population. By applying FLI into clinical practice, general practitioners could improve the detection of IHD.

The findings from our present study supported our assumption that the FLI level is associated with the prevalent IHD in the general population. The first step of our analysis focused on the association between FLI level and the prevalent IHD via the logistic regression analysis. In the multivariate-adjusted model, our results demonstrated a significant and positive association between FLI level and the prevalent IHD. The Model 2 of Table 2 was adjusted for demographic, anthropometric, laboratory, and medical history covariates. Therefore, the association between FLI and prevalent IHD was independent of the conventional cardiovascular risk factors. However, the logistic regression model was conducted under the hypothesis that the association between FLI and prevalent IHD was linear in the whole range of FLI. If the actual relationship is non-linear, the logistic regression results will deviate from the actual relationship, thereby giving us the wrong information. To address this question, we employed a smooth curve fitting (conducted by GAM) and a logarithmic likelihood ratio test in step two of our statistical analysis. The results displayed that the association between normalized FLI level and the prevalent IHD was positively linear in the full range of FLI. Therefore, the ratio of prevalent IHD may increase proportionally with the increment of FLI level in the full range of FLI, and there may be no threshold or saturation effect in their association.

To evaluate whether our main finding was consistent in conventional cardiovascular subgroups, we conducted a subgroup analysis. The results demonstrated no significant interaction between the grouping variates (including age, sex, race, BMI, hypertension, and diabetes) and the association between FLI and IHD. However, we also observed a trend towards a larger effect size (OR value) in female subjects and diabetes patients. The association of FLI and prevalent IHD could be more prominent in female and diabetes populations. And the insignificance of the interaction effect among sex and diabetes subgroups could be due to a lack of statistical power. Therefore, more studies with larger sample sizes are needed to confirm our observation. In general, our main result is still effective in these subpopulations; applying the relationship between FLI and the prevalent IHD in these subpopulations is reasonable, and in female subjects and diabetes patients, the association could be more prominent.

With a clear depiction of the association between FLI and the prevalent IHD, the fourth step of our analysis shifted the focus to the potential value of FLI to improve the detection of prevalent IHD in the general population. We used ROC and reclassification analysis to evaluate the novel index in this step. In ROC analysis, the AUC of FLI alone for recognizing prevalent IHD was limited. Therefore, using FLI alone in clinical practice will achieve a satisfying result. However, when introducing FLI into conventional cardiovascular risk factors, the entire model significantly improved the detecting ability of prevalent IHD. These findings suggest the potential value of FLI to optimize the detection of the prevalent IHD in the general population. Although the ROC analysis is the most popular approach to evaluate the value of a novel marker, we noticed that it concentrated on the integral ability of the entire model to detect prevalent conditions or diseases. Specifically, ROC analysis actually assesses the capability of the entire model (conventional cardiovascular risk factors + FLI) to identify the prevalent IHD rather than investigate the value of FLI itself to optimize the detection of prevalent IHD. ROC analysis could overestimate or underestimate the potential of FLI (19). Therefore, the results merely from ROC analysis could not provide accurate information about whether introducing FLI into conventional cardiovascular risk factors would make the detection of the prevalent IHD more accurate (20). To evaluate the value of FLI at an angle different from ROC analysis, statisticians have put forward the reclassification analysis, including NRI and IDI (21–23). In the present study, after adding FLI into conventional cardiovascular risk factors, both continuous NRI and IDI revealed a significant improvement in detecting the prevalent IHD. Therefore, combining FLI with conventional cardiovascular risk factors will reclassify more subjects into the actual categories (IHD or non-IHD). In general, the results from both ROC and reclassification analysis suggest that applying FLI could help optimize the detection of prevalent IHD in the general population.

Our findings were consistent with the results from two previous articles. Olubamwo et al. recruited 501 subjects without cardiometabolic disease (type 2 diabetes or cardiovascular disease) to assess the association between FLI and the risk of developing cardiometabolic diseases during a mean follow-up of 15 years. Their results demonstrated that persons with significant FLI increase will likely have an increasing cardiometabolic disease risk (24). Kim et al. employed the data from 3011,588 Korean to evaluate the usefulness of FLI in predicting major adverse cardiac events (MACEs, including IHD events) during a median follow-up of 6 years (14). Their results demonstrated a linear association between higher FLI values and higher incidence of the MACEs. Our study showed some differences with their work. Firstly, their studies focused on the value of FLI in predicting the development of cardiometabolic diseases or MACEs, and neither study conducted a specified subgroup regarding IHD. Meanwhile, our work was intended to investigate the potential of FLI to detect the presence of IHD in the general population. Therefore, the findings from our work and their studies supported the usefulness of FLI in different application conditions, our work suggested the value of FLI as a detection marker of IHD, and their studies implicated the value of FLI as a prediction index for the risk of developing cardiometabolic diseases or MACEs. Secondly, Olubamwo et al.'s study did not assess whether the association between FLI and outcomes was linear; Kim et al.'s study only evaluated the linearity by dividing FLI into deciles without any statistical test, which is relatively rough. On the contrary, our study employed a smooth curve fitting analysis and a logarithmic likelihood ratio test to investigate the linearity of the association between FLI and the prevalent IHD. Thirdly, both studies only provided the effect size of the associations between FLI and outcomes, but did not give information about the performance of FLI in ROC analysis. Our current study presented the ROC results and conducted the reclassification analysis to assess the value of FLI to detect prevalent IHD from a different angle from ROC. Lastly, our study population also showed differences from their populations. Different lifestyles, diet habits, geographic and socioeconomic conditions could impact the association between FLI and outcomes.

Although similar, the current study differed from our previously published article (25). The current study discovered that FLI, and the underlying severity of NAFLD, are associated with the prevalent IHD. FLI could improve the detection of IHD in the general population. While in the previous study, we focused on the value of the weight-adjusted waist index in identifying prevalent HF in the general population. The current study focused on IHD, while the previous research focused on HF, the target disease differs between the two studies. As for the additional contribution of the current study, we identified a potential biomarker to improve the detection of IHD, which could improve cardiovascular health in the general population. Furthermore, the previous research focused on the impact of excessive fat accumulation on cardiovascular health, while the current study pays attention to the value of monitoring the severity of NAFLD.

There are multiple mechanisms behind the association between NAFLD and increased risk of IHD (26). Firstly, endothelial dysfunction was observed in NAFLD (27). NAFLD patients exhibit an elevated level of circulating ADMA, which is an endogenous antagonist of nitric oxide synthase and is positively associated with several cardiovascular diseases (28). Besides, other markers of endothelial dysfunction are also increased in NAFLD patients (29, 30). Disruption and dysfunction of the endothelial layer play a role in atherogenesis and subsequent cardiovascular diseases. Secondly, serum homocysteine is reported to be increased in NAFLD (31). Alteration of homocysteine metabolism results in increased burden of oxidative stress, which is generally increased in NAFLD (32). Oxidative stress is essential in cardiovascular pathophysiology (33). Additionally, cytokines released by the diseased liver drain into the systemic circulation, resulting in consequential cardiovascular effects. Systemic inflammation and circulating cytokines, such as interleukin 1, interleukin 6 and tumor necrosis factor α, are associated with cardiovascular diseases (34, 35). Thirdly, the lipid profile is significantly changed in NAFLD. Increased TG and LDL-c levels, decreased HDL-c level, and other changes in lipid components synergistically lead to more atherogenic lipid profiles (36, 37). Lastly, other mechanisms like arterial structural alterations, hepatokines, adipokines, Gut-liver axis, angiogenic factors, and genetic factors also play their roles in the mechanism underlying the association between NAFLD and IHD (26).

It is necessary to mention the limitations when interpreting our results. Firstly, due to the nature of the cross-sectional design of NHANES, our results could only provide a clue for the association between FLI and the prevalent IHD, as well as the potential value of FLI to improve the detection of prevalent IHD in the general population. Secondly, the detection of IHD in our analysis was based on the subjects’ self-report. Therefore, the accuracy of the detection was limited. Nevertheless, the NHANES study was conducted according to standard operating procedures. The result from the questionnaire is still reliable. Thirdly, the findings of the current analysis were based on a general population in America. Therefore, whether these findings possess external applicability to the population with a different lifestyle, diet habit, geographic and socioeconomic conditions remain unclear. Fourthly, Although the *P* for non-linearity showed insignificance, Figure 1 showed that the risk for prevalent IHD increased more rapidly with the elevation of FLI in the region of normalized FLI > 1 than in the region of normalized FLI < 1. We speculate that this phenomenon could be due to a lack of statistical power in this region. Therefore, more studies with larger sample sizes are needed to confirm this phenomenon. Fifthly, we observed a larger OR for the association between FLI and prevalent IHD in female subjects than in male subjects, and a larger OR for the association in diabetes patients than in non-diabetes subjects. However, due to the limited statistical power, the interaction effects of sex and diabetes did not achieve significance. Therefore, the association between FLI and prevalent IHD could be more prominent in females and diabetes patients, and more studies with larger sample sizes are needed to confirm our observation. Lastly, the same as other observational research, residual confounding caused by some unincluded covariables could lead to bias in our results. For example, as we mentioned before, homocysteine and cytokines like interleukin 1, interleukin 6, and tumor necrosis factor *i* play their roles in the association between NAFLD and IHD, but these variates were not collected in our current survey. Based on the above points, a long-term and prospective study with a more reliable IHD definition and more detailed information collection is warranted to confirm our findings in the future.

## Data Availability

The datasets presented in this study can be found in online repositories. The names of the repository/repositories and accession number(s) can be found below: https://wwwn.cdc.gov/nchs/nhanes/continuousnhanes/default.aspx?BeginYear=1999.
